# Comprehensive Case–Control Study of Protective and Risk Factors for Buruli Ulcer, Southeastern Australia

**DOI:** 10.3201/eid2910.230011

**Published:** 2023-10

**Authors:** Bridgette J. McNamara, Kim R. Blasdell, Arvind Yerramilli, Ina L. Smith, Simone L. Clayton, Michael Dunn, Ee Laine Tay, Katherine B. Gibney, Nilakshi T. Waidyatillake, Mohammad A. Hussain, Michael Muleme, Daniel P. O’Brien, Eugene Athan

**Affiliations:** Barwon Health, Geelong, Victoria, Australia (B.J. McNamara, A. Yerramilli, M.A. Hussain, M. Muleme, D.P. O’Brien, E. Athan);; University of Melbourne, Melbourne, Victoria, Australia (B.J. McNamara, K.B. Gibney, N.T. Waidyatillake, D.P. O’Brien);; Commonwealth Scientific and Industrial Research Organisation, Geelong (K.R. Blasdell, S.L. Clayton, M. Dunn);; Commonwealth Scientific and Industrial Research Organisation, Canberra, Australian Capital Territory, Australia (I.L. Smith);; Department of Health, Melbourne (E.L. Tay);; Peter Doherty Institute for Infection and Immunity, Melbourne (K.B. Gibney);; Deakin University, Waurn Ponds, Victoria, Australia (N.T. Waidyatillake, E. Athan)

**Keywords:** Buruli ulcer, *Mycobacterium ulcerans*, *Mycobacterium*, risk factors, protective factors, case-control studies, bacteria, tuberculosis and other mycobacteria, vector-borne infections, zoonoses, Australia

## Abstract

To examine protective and risk factors for Buruli ulcer (BU), we conducted a case–control study of 245 adult BU cases and 481 postcode-matched controls across BU-endemic areas of Victoria, Australia. We calculated age- and sex-adjusted odds ratios for socio-environmental, host, and behavioral factors associated with BU by using conditional logistic regression. Odds of BU were >2-fold for persons with diabetes mellitus and persons working outdoors who had soil contact in BU-endemic areas (compared with indoor work) but were lower among persons who had bacillus Calmette–Guérin vaccinations. BU was associated with increasing numbers of possums and with ponds and bore water use at residences. Using insect repellent, covering arms and legs outdoors, and immediately washing wounds were protective; undertaking multiple protective behaviors was associated with the lowest odds of BU. Skin hygiene/protection behaviors and previous bacillus Calmette–Guérin vaccination might provide protection against BU in BU-endemic areas.

Buruli ulcer (BU) is a necrotizing infection of the skin and soft tissue caused by the environmental bacterium *Mycobacterium ulcerans* (*1*,*2*) and is 1 of 20 neglected tropical diseases recognized by the World Health Organization (*3*). BU often begins as a small papule or plaque with progressive ulceration if left untreated (*4*). The incubation period is ≈4–5 months, whereas the average delay from symptom onset to diagnosis is 1–2 months (*5*–*7*). Although sporadic cases have been noted globally, BU remains endemic in sub-Saharan Africa and more temperate southeastern Australia, 2 regions with vastly differing social and environmental conditions (*8*). In southeastern Australia, cases are most frequently detected in Mornington and Bellarine Peninsulas, regions on opposite sides of Port Philip Bay in Victoria state (*6*). BU case numbers have increased markedly in the previous decade in Victoria; disease-endemic areas within the region have expanded (*9*,*10*), but the reasons remain unclear.

The exact mechanisms of *M. ulcerans* transmission are elusive and might differ between endemic areas. Nevertheless, research has revealed certain key variables; leading theories involve insect bites or environmental contamination through minor trauma or existing wounds (*2*,*11*). In southeastern Australia, possums evidently play a crucial role as an animal reservoir that can sustain clinical disease and shed viable *M. ulcerans* through feces (*12*–*14*). Two species in particular, the common brushtail (*Trichosurus vulpecula*) and common ringtail (*Pseudocherius peregrinus*) possums, have been implicated as reservoir hosts. Furthermore, research in Australia reports mosquitoes as possible mechanical vectors (*15*–*17*). A previous questionnaire-based case–control study in Victoria showed that being bitten by mosquitoes increased the odds of *M. ulcerans* infection, whereas wearing protective clothing or applying insect repellent decreased the odds (*18*). In contrast, no convincing evidence exists that mosquitoes play a role in *M. ulcerans* transmission in West Africa. *M. ulcerans DNA* has been detected in environmental samples of other insects from aquatic areas in West Africa, such as water bugs (Hemiptera), dragonfly larvae (Odonata), and beetle larvae (Coleoptera) (*2*).

Environmental and climate factors also appear to play a critical role in *M. ulcerans* transmission dynamics. In Africa, cases of BU occur proximate to natural water bodies (*2*). Heavy rainfall and subsequent flooding have also been associated with increased detection of *M. ulcerans* in the environment and increased BU case numbers in certain regions (*9*,*19*). Environmental surveys, conducted as a separate part of this research project, showed that the odds of *M. ulcerans* bacteria existing within a property increased with the presence of certain native plant species, alkaline soil, and lower altitude, along with the presence of overhead powerlines and common ringtail possums (*14*).

Cleaning wounds immediately after trauma and the use of *Mycobacterium bovis* bacillus Calmette–Guérin (BCG) vaccination (for tuberculosis, also caused by a mycobacterium) might mitigate the risk of acquiring BU, although evidence regarding BCG vaccination is conflicting (*18*,*20*,*21*). In addition, BU lesions are common on exposed body areas, consistent with the premise that protective clothing might decrease BU risk by reducing insect bites and minor skin trauma that can cause potential inoculating events (*22*,*23*).

Determining risks and protective factors for BU is crucial to determine effective intervention and control strategies. Therefore, we conducted a case–control study to identify environmental, host, and behavioral risk and protective factors associated with BU in Victoria, Australia, where increasing cases and expanding BU-endemic areas have been observed.

## Methods

### Study Design and Participants

**Figure 1:**
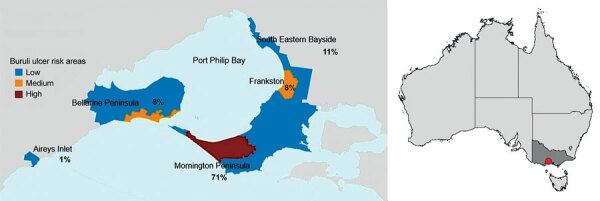
Locations of Buruli ulcer–endemic areas included in comprehensive case-control study of protective and risk factors for Buruli ulcer, Victoria, Australia. Colors indicate risk classifications at beginning of the study period, and numbers indicate percentage of total participating case-patients for each location within the study area. Full map of Australia shows study area in southeastern region.

**Figure 2:**
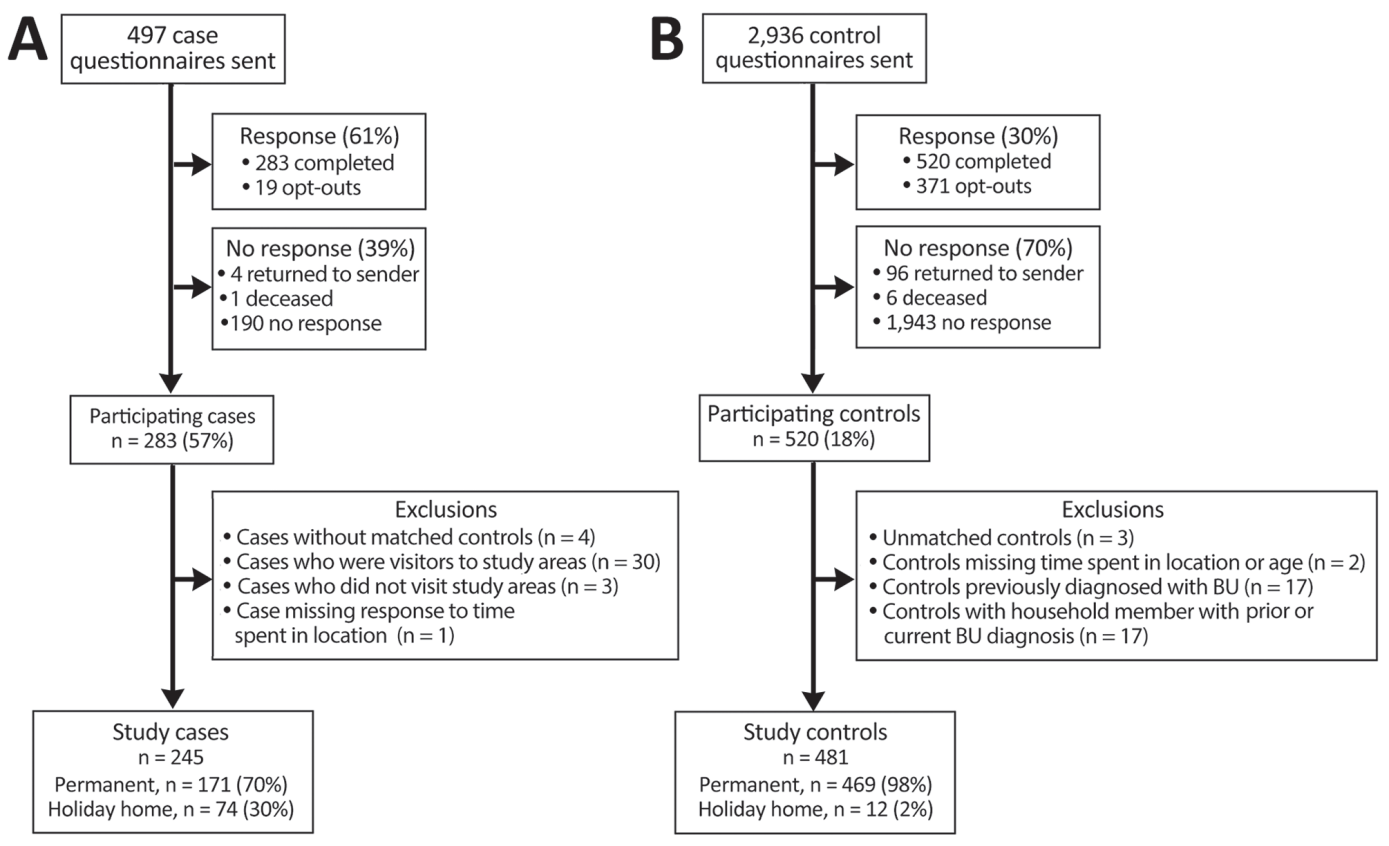
Flow diagrams of study recruitment, participation, and exclusion criteria in comprehensive case–control study of protective and risk factors for Buruli ulcer, southeastern Australia. A) Case-patient recruitment; B) control recruitment.

We performed a postcode matched, case–control study in BU-endemic areas surrounding Port Phillip Bay, Victoria, Australia (Figure 1; Appendix Table 1). Ethics approval was granted by the Victoria Department of Health Human Research Ethics Committee (project 10–18). We invited adults (>18 years of age) to participate in the study who resided in Victoria and were notified to the Department of Health in Victoria as having laboratory-confirmed BU during June 2018–June 2020. We extracted case data from the Victoria Department of Health Public Health Events Surveillance System. We recruited case-patients via regular mail after receiving permission for contact from the patient’s general practitioner or treating medical team. We restricted analysis to residents or holiday homeowners in the study areas (Figure 2).

We matched control participants (residents of Victoria >18 years of age) to patients according to residential postal codes within the study area. We selected controls from both the Victorian Population Health Survey (participants who had provided consent to be contacted for other research studies) and the electoral roll of Australia (when additional matched controls were required for a particular postcode). We excluded controls if they or a household member had been previously diagnosed with BU (Figure 2).

Participation for both patients and controls involved the return of a completed study questionnaire. In addition, a subsample of patients and controls were enrolled in an environmental survey of residential properties that investigated the presence of *M. ulcerans* (*14*).

### Data Collection and Measurements

We used a self-administered questionnaire to examine the amount of time participants spent in the study areas, outdoor and lifestyle behaviors, insect exposure, medical history, and environmental characteristics of the participants’ properties. We evaluated those details and formulated response and collapsed categories for analysis (Appendix Table 2). Participant-reported medications and conditions that might affect the immune system were reviewed by a physician specializing in infectious diseases (D.P.O.) to ascertain those likely to cause immunosuppression. We devised an occupational classification related to potential environmental exposure to *M. ulcerans* through employment by using participant responses to 2 questions: what proportion of your time do you spend outside as part of your occupation and are you in contact with the soil during your work? We examined the effects of working outdoors and having soil contact among participants whose employment was based in the study (disease-endemic) areas only.

### Statistical Analysis

We evaluated host, environmental, and behavioral factors according to BU case status. We examined relationships between those factors and the likelihood of developing BU by using multivariable conditional logistic regression; cases and controls were matched by postcode. We calculated odds ratios adjusted for age and sex (aORs) and 95% CIs for the total participant sample (residents and holiday homeowners) and separately for residents only (Appendix Tables 3–11). Percentages of missing data were generally low (<3% for most factors); if missing data were >10%, we included a separate category for those participants with missing exposure data in the model unless otherwise stated. Given the expectation that participants might have multiple potentially protective health behaviors, we examined patterns and clustering of those behaviors by using polychoric correlations and exploratory factor analysis (Appendix Figures 2, 3).

We conducted a post-hoc sensitivity analysis to explore the robustness of the observed relationship between BCG vaccination and BU case status; we restricted analysis to participants 47–70 years of age who were within the age-range eligible for BCG vaccination as part of the routine vaccination schedule for schoolchildren in Victoria from the 1950s to 1985 (*24*). We analyzed those reporting receipt of BCG vaccination and those unsure of vaccination status as a single category (under the assumption of likely vaccination through routine vaccination) and compared them with age-matched participants reporting no BCG vaccination. We performed analyses by using Stata 15 (StataCorp LLC, https://www.stata.com) except for factor analysis, which we performed by using Stata 16.

## Results

### Demographic and Clinical Characteristics of Participants

We examined data from 245 (57% participation rate) BU case-patients and 481 (18%) postcode-matched control participants from across the BU-endemic areas; 171 (70%) patients and 469 (97.5%) controls were permanent residents in the study areas, and most (71%) were homeowners in high BU-endemic areas of Mornington Peninsula (Figure 1). Half (123/245) of case-patients were 60–79 years of age, signifying an overrepresentation when compared with all notified cases in the study areas (204/550 [37%] 60–79 years of age). In contrast, patients 18–39 years of age were underrepresented in our participant sample (35/245 [14%] compared with 134/550 [24%] among notified cases) (Appendix Table 12). We also observed an overrepresentation of controls 60–79 years of age and a large underrepresentation of controls 18–39 years of age when compared with population proportion estimates (Appendix Table 12). Male sex was associated with BU case status (57.6% of BU cases vs. 44.7% of controls; aOR 1.52 [95% CI 1.06–2.19]).

BU cases were reported predominantly during winter (44%) and spring (38%) (Table; Appendix Figure 1). The median time between symptom onset and diagnosis was 5 (interquartile range [IQR] 3–12) weeks; duration was longer for patients who were holiday homeowners (8 [IQR 4–13]) weeks than for those who were residents (4 [IQR 3–10] weeks; p<0.0001 by rank-sum test). An insect bite, wound, or injury to the affected area was reported in 36% of BU cases before ulcers appeared.

**Table:** Characteristics of patients and disease manifestations in comprehensive case–control study of protective and risk factors for Buruli ulcer, southeastern Australia*

Characteristics	Cases, n = 245	Controls, n = 481
Age group, y		
18–39	35 (14)	38 (8)
40–59	68 (28)	125 (26)
60–79	123 (50)	278 (58)
>80	19 (8)	40 (8)
Sex		
F	104 (42)	266 (55)
M	141 (58)	215 (45)
Employment status†		
Employed	124 (51)	211 (44)
Unpaid employment, unemployed	19 (8)	18 (4)
Retired	100 (41)	249 (52)
Notification dates		
Summer, Dec–Feb	26 (11)	NA
Autumn, Mar–May	18 (7)	NA
Winter, Jun–Aug	107 (44)	NA
Spring, Sep–Nov	94 (38)	NA
Duration of symptoms before diagnosis, wk		
Median (IQR)	5 (3–12)	NA
Missing data	21 (9)	NA
Days from notification to questionnaire completion		
Median (IQR)	56 (38–90)	NA
Insect bite/wound/injury to area before ulcer developed		
Yes	99 (40)	NA
No	42 (17)	NA
Unsure	88 (36)	NA
Missing data	16 (7)	NA
Type of bite/wound/injury in area before ulcer developed, n = 99		
Insect bite	51 (52)	NA
Wound/injury	30 (30)	NA
Mixed	6 (6)	NA
Other, unsure/missing data	12 (12)	NA
Time from wound/bite to ulcer, if yes, n = 87		
Median, weeks (IQR)	6 (3–13)	NA

*Values are no. (%) except as indicated. IQR, interquartile range; NA, not applicable. †Unpaid employment included students and persons with home duties.

### Host Factors

**Figure 3:**
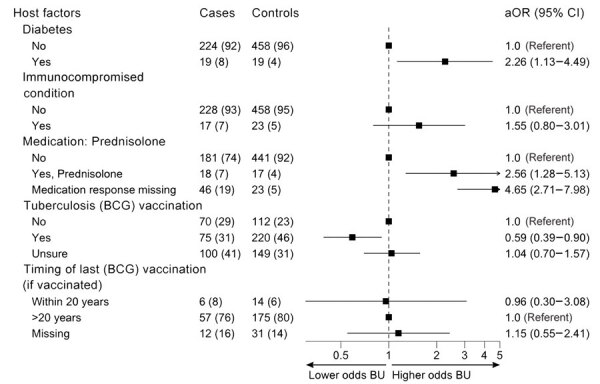
Odds of developing Buruli ulcer according to different host factors in comprehensive case–control study of protective and risk factors for Buruli ulcer, southeastern Australia. Host characteristics are shown for case-patients and control participants as no. (%). Odds ratios (adjusted according to age and sex) and 95% CIs are indicated. Vaccination was with *Mycobacterium bovis* BCG vaccine for tuberculosis. Immunocompromised conditions category was for any participant who reported a condition that had the potential to compromise the immune system (excluding diabetes and cancer [active or historical]; cancer status was not available in this study). aOR, adjusted odds ratio; BCG, bacillus Calmette-Guérin vaccine; BU, Buruli ulcer.

We evaluated associations between host factors and BU case status (Figure 3). Persons with a history of diabetes mellitus had a higher probability of developing BU than those without diabetes (aOR 2.26 [95% CI 1.13–4.49]). An association was observed with prednisolone therapy (aOR 2.56 [95% CI 1.28–5.13]); however, this result could be confounded by persons commencing prednisolone therapy during their BU treatment.

Receipt of BCG vaccination was associated with lower odds of BU (aOR 0.59 [95% CI 0.39–0.90]) than for participants reporting no BCG vaccination. No relationship between BU and vaccination timing (<20 or >20 years ago) was observed. Of note, 41% of patients and 31% of controls reported that they were unsure whether they had received the vaccination. In the sensitivity analysis that restricted participant age to 47–70 years (those unsure were assumed vaccinated), the observed association between BU and BCG vaccination persisted but was attenuated; aOR was 0.71 (95% CI 0.41–1.22) for the entire age-restricted participant sample (Appendix Table 11).

### Environmental Factors

**Figure 4:**
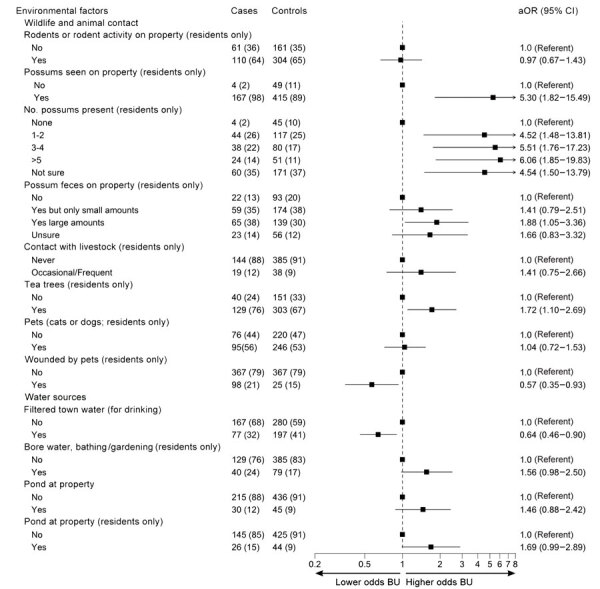
Odds of developing Buruli ulcer according to different environmental factors in comprehensive case–control study of protective and risk factors for Buruli ulcer, southeastern Australia. Environmental factors are shown for case-patients and control participants as no. (%). Odds ratios (adjusted according to age and sex) and 95% CIs are indicated. aOR, adjusted odds ratio; BU, Buruli ulcer.

The presence of possums around the property was strongly associated with BU in residents (aOR 5.30 [95% CI 1.82–15.49]) and, to a lesser extent, in the entire participant sample (aOR 2.33 [95% CI 1.15–4.71]). The likelihood of developing BU increased with the number of possums reported around the residential property (Figure 4; Appendix Table 5); large amounts of possum feces (compared with none) (aOR 1.88 [95% CI 1.05–3.36]); and with the presence of tea trees (*Leptospermum* sp.), a common habitat for possums, on the property (aOR 1.72 [95% CI 1.10–2.69]).

Most (98%) properties used piped (town) water for drinking, bathing, and garden watering. Participants drinking filtered town water (274/721, 38% of total participants) had lower odds of developing BU than those not drinking filtered town water (aOR 0.64 [95% CI 0.46–0.90]). Of those not drinking filtered town water, 433/447 (97%) drank unfiltered town water, and 14 (3%) drank water from other sources only, such as tank or bottled water. Use of bore water by residents for bathing or garden watering was associated with BU (aOR 1.56 [95% CI 0.98–2.50]). Water sources around the property were not associated with BU case status, except for the presence of ponds (aOR 1.69 [95% CI 0.99–2.89]) for residents (Figure 4). We observed no associations between case status and the presence of other nonpossum wildlife or biting insects; use of garden products (mulch or potting mix) among residents; or with earthworks, major renovations, or sewerage works near the property (Appendix Table 7).

### Exposures

**Figure 5:**
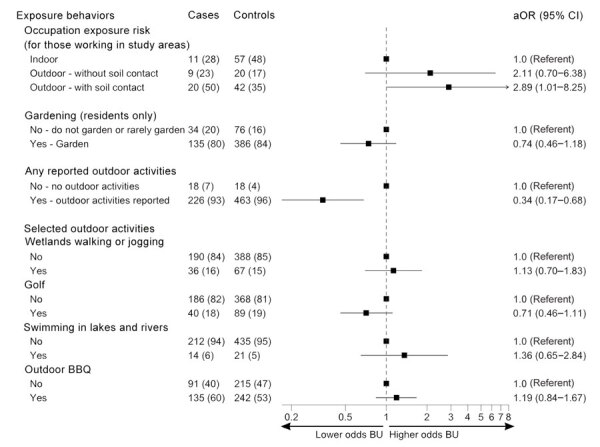
Odds of developing Buruli ulcer according to potential outdoor exposures in comprehensive case–control study of protective and risk factors for Buruli ulcer, southeastern Australia. Potential outdoor exposures are shown for case-patients and control participants as no. (%). Odds ratios (adjusted according to age and sex) and 95% CIs are indicated. aOR, adjusted odds ratio; BBQ, barbeque; BU, Buruli ulcer.

Working outdoors was associated with higher odds of BU than working indoors in BU-endemic areas (Figure 5); highest odds were associated with occupations involving soil contact (aOR 2.89 [95% CI 1.01–8.25]). Outdoor occupations that involved soil contact were commonly gardeners, carpenters, and other construction-related roles.

We found no association between gardening frequency and BU case status among residents (Figure 5); however, the entire participant sample comprising more holiday homeowner cases had lower odds for BU (aOR 0.50 [95% CI 0.34–0.74]). Participants partaking in outdoor activities (>95% of participants) had a lower likelihood of developing BU than those not undertaking outdoor activities (aOR 0.34 [95% CI 0.17–0.68]). However, we observed no strong associations between participants undertaking individual activities (beach walks/jogging, wetland walks/jogging, bushwalking, golf, sports on an oval, swimming in local lakes/rivers, sailing, outdoor barbeques, or other activities) and those not undertaking the activity (Appendix Table 9).

### Protective Behavioral Factors

**Figure 6:**
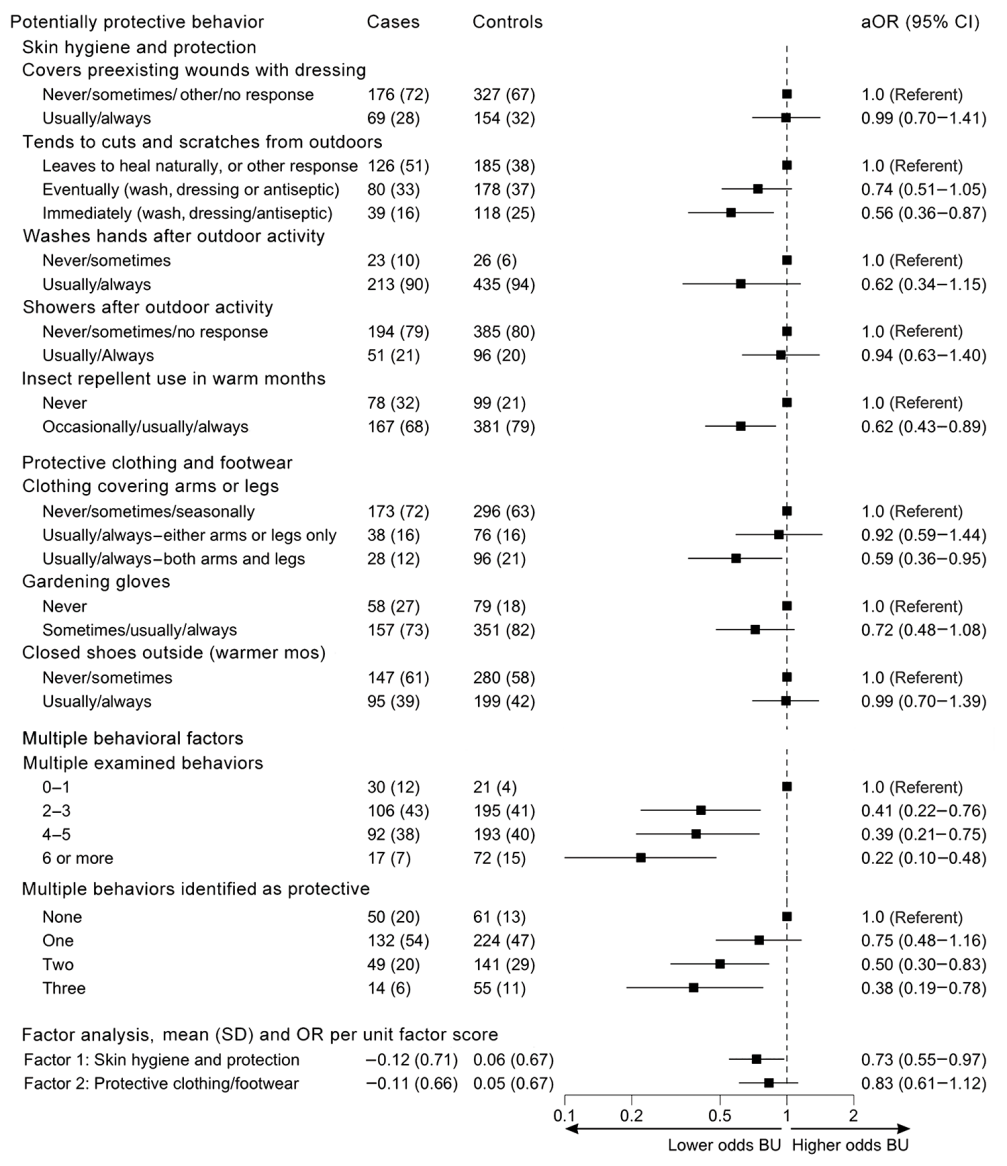
Odds of developing Buruli ulcer according to protective behavioral factors in comprehensive case-control study of protective and risk factors for Buruli ulcer, southeastern Australia. Potential protective behavioral factors are shown for case-patients and control participants as no. (%), except for factor analyses, which are shown as mean (SD). Odds ratios (adjusted according to age and sex) and 95% CIs are indicated. Includes binary variable for tending to outdoor cuts and scratches immediately (usually/always vs all other responses). aOR, adjusted odds ratio; BU, Buruli ulcer; OR, odds ratio.

We analyzed associations between protective health behaviors and BU case status (Figure 6). Several protective behaviors were associated with lower odds of developing BU: tending immediately to cuts and scratches received during outdoor activity by washing the area and then applying antiseptic or dressings (aOR 0.56 [95% CI 0.36–0.87]), wearing insect repellant during warmer months (aOR 0.62 [95% CI 0.43–0.89]), and covering arms and legs with clothing (aOR 0.59 [95% CI 0.36–0.90]). Participants who combined protective behaviors had the strongest correlations between tending to new wounds, covering preexisting wounds, washing hands after outdoor activity, and using gloves for gardening (Appendix Figures 2, 3). Combining protective behaviors was associated with lower odds of BU; we observed a gradient of decreasing odds for BU in those undertaking higher numbers of protective behaviors (Figure 6).

## Discussion

We conducted a comprehensive case–control study in temperate, BU-endemic areas of Victoria, Australia, and found that the presence of possums or a pond on residential property was a key environmental factor for BU, whereas having diabetes mellitus and working outdoors (especially in contact with soil) were key host factors associated with higher probability of developing BU. We identified modifiable health behaviors for public health intervention relating to skin hygiene and protection, including tending immediately to outdoor cuts and scratches by cleaning and applying antiseptic or dressing, using insect repellant, and covering arms and legs with clothing. Moreover, undertaking multiple protective behaviors was associated with lower odds of developing BU. We found a protective association between BCG vaccination and BU, as well as the unexpected finding of a protective association for drinking filtered town water compared with unfiltered water, which warrants further investigation. We found no evidence for associations between BU and other hypothesized risks, including gardening, other outdoor leisure activities, pet ownership, major renovations or earthworks, or sewerage type or works.

Our findings strengthen the evidence for possums as a key mammal reservoir of *M. ulcerans* in Victoria (*12*,*14*). Possums can become infected with *M. ulcerans*; >40% of possum fecal samples collected in 1 BU-endemic area were positive for *M. ulcerans* DNA, and a considerable proportion of possums displayed BU skin lesions (*12*). The environmental survey component of this study found possum feces to be a key source of viable bacteria (*14*); *M. ulcerans* DNA was found in 23% and viable *M. ulcerans* bacteria in 5% of all ringtail possum fecal samples (*14*). According to participant responses, we found that increased likelihood of BU was associated with increasing numbers of possums at the participant’s property and with increasing amounts of possum feces. The number of tea trees, a common possum habitat, on the property was also highly associated with BU case status.

The involvement of aquatic environments has been suggested for *M. ulcerans* transmission in BU-endemic areas of West Africa, but limited evidence has been found in Victoria (*2*,*11*). In our study, residential ponds and use of bore water were associated with BU. Contributions to BU incidence remain unclear for direct contact with contaminated water; ponds providing habitat for mosquitoes, which could act as mechanical vectors; or ponds attracting mammal reservoirs. The protective association found for piped, filtered town drinking water was unexpected; town water catchments for BU-endemic areas also provide water to many nonendemic metropolitan areas; thus, the protective association for water filtration might reflect other unmeasured confounding factors affecting BU risk. Furthermore, correlations between drinking filtered water and other potentially protective behaviors were relatively weak (correlation coefficient <0.18), and clustering of those behaviors does not appear to explain the association. Although *M. ulcerans* infection in the gastrointestinal tract of infected possums has been reported (*25*), whether *M. ulcerans* exposure via ingestion could result in BU skin lesions in humans is unclear. The relationship between bore water and BU might not indicate bore water use is a risk factor for BU; rather, bore water might be associated with the presence of *M*. *ulcerans* in the environment, such as in plants or possums. 

Mosquitoes have been proposed as likely mechanical vectors for BU in Australia but are less likely candidates in West Africa (*11*). We did not find associations between reported levels of local mosquitoes or other biting insects and BU. However, we did find a protective association between BU and use of insect repellant, consistent with a previous case–control study on Bellarine Peninsula in Victoria, where 72% lower odds of BU were found among persons using insect repellent (*18*). In contrast to that study, we found a relatively higher percentage of persons reporting insect repellent use (68% vs. 31% of case-patients and 79% vs. 54% of controls). Our results indicate a positive public health development, given the role of mosquitoes in transmission of several arboviral diseases, and might be the result of local public health campaigns (*10*), such as Beat the Bite (https://www.betterhealth.vic.gov.au/sites/default/files/2021-10/Beat-the-bite-brochure.pdf).

Skin protection and skin hygiene behaviors were associated with lower odds of BU. We found that tending to cuts and scratches during outdoor activity by stopping immediately to wash the area and applying antiseptic or a dressing had the strongest protective association, which is consistent with previous studies in Australia (*18*) and Cameroon (*26*). However, our study adds new evidence suggesting a dose-response association that indicates the timeliness of tending to wounds might also help prevent BU; lower odds of BU were observed for immediate treatment compared with leaving the wound alone or tending eventually. Cuts and scratches obtained during outdoor activities or work might increase inoculating events with *M. ulcerans*, which might be present on the skin after contact with contaminated soil, plants, or water. Laboratory studies have demonstrated that a needle puncture or mosquito bite on contaminated skin was sufficient for *M. ulcerans* to enter the skin of mice and cause an ulcer (*15*). In our study, bites or wounds were reported in 40% of cases before ulcer appearance; some participants recalled specific injuries to the area that preceded ulcer development.

The higher odds of BU in persons with diabetes is similar to findings for other mycobacterial diseases, such as tuberculosis and leprosy (*27*), and might reflect increased risk because of impaired cellular immunity (*28*). Targeted messaging highlighting the importance of protective measures might help prevent BU in persons with diabetes.

We showed that BCG vaccination was highly protective against BU (aOR 0.59 [95% CI 0.39–0.90]). Protective effects of BCG vaccination against tuberculosis and leprosy have been well established (*29*). The vaccine is derived from a live attenuated strain of *M*. *bovis* and shares epitopes with other nontuberculous mycobacteria (*20*). Previous case–control studies showed conflicting evidence that BCG vaccination prevents *M. ulcerans* infection (*29*–*32*). Two randomized controlled trials demonstrated a protective effect of BCG vaccination against BU (*33*,*34*); a lower incidence of BU in persons vaccinated with BCG compared with unvaccinated persons was observed in Uganda, with a combined relative risk estimate of 0.50 (95% CI 0.37–0.69) (*20*). However, both of those studies demonstrated only short-term efficacy up to 1 year after vaccination; longer-term follow up and analysis were not performed because of limited sample size. Using different antigenic strains of BCG might enhance or lengthen protection against nontuberculous mycobacteria or BU (*20*,*29*), whereas revaccination could also provide more sustained immunity to *M. ulcerans* infection, although this idea has not been comprehensively explored (*20*). Further research on the potential role of BCG vaccination for protection against BU is warranted.

A key strength of our study of BU risk factors is the use of a population-based notifiable disease database for case detection that ensured robust ascertainment of laboratory-confirmed BU from almost all BU-endemic locations in Victoria. Compared with a previous case–control study in the Bellarine Peninsula, Victoria (*18*), this study also examined a comprehensive list of environmental, host and behavioral risk, and protective factors, and we have identified new public health-related risk groups and environmental risk factors. The graded responses observed for certain individual protective behaviors as well as multiple combined behaviors offers strong evidence and support for causal inference despite the limitations of the observational study design.

The first limitation of our study is the potential for recall bias given the long disease incubation period, potential for differential recall if patients were more aware of hypothesized transmission pathways than controls, and potential effects of seasonality on recall by matched controls who were recruited after the patients. Second, potential selection bias was noted because of differential participation between patients and controls; younger patients were more likely to participate than younger control participants, and a greater proportion of holiday homeowners existed among BU cases. Despite those limitations, survey completion in this study was rapid (within 2 months of diagnosis for most cases) compared with the previous case–control study in Victoria (*18*), which had a median completion rate of 1.5 years postdiagnosis. We adjusted all analyses for age and sex, and the postcode-matched design helped account for unmeasured socioeconomic and environmental differences across the BU-endemic areas. By analyzing results for the entire cohort and separately for residents only, we found strong associations among the resident cohort and differential effects of home ownership. Finally, our findings are relevant to Victoria, Australia, and might offer insights relevant to other areas; however, those data might not be immediately generalizable to other parts of the world.

In conclusion, our study identifies environmental and host factors associated with BU and simple behaviors relating to skin hygiene and protection that appear to mitigate the risk of developing BU. We highlight areas that warrant further investigation, particularly the potential role of the BCG vaccine in mitigating BU risk. Our findings are essential to inform public health strategies for BU prevention, especially for persons at highest risk in BU-endemic areas who work outdoors and those with diabetes.

Appendix Additional information for comprehensive case–control study of protective and risk factors for Buruli ulcer, southeastern Australia.
