# Supplementary material for: Comprehensive Case–Control Study of Protective and Risk Factors for Buruli Ulcer, Southeastern Australia
Source: Emerg Infect Dis. 2023 Oct;29(10):2032–43. doi: 10.3201/eid2910.230011 (PMC10521623; doi:10.3201/eid2910.230011)
Supplement: Appendix — Additional information for comprehensive case–control study of protective and risk factors for Buruli ulcer, southeastern Australia. [file 23-0011-Techapp-s1.pdf]

Article DOI: https://doi.org/10.3201/eid2910.230011

EID cannot ensure accessibility for supplementary materials supplied by authors. Readers who have difficulty accessing supplementary content should contact the authors for assistance.

Comprehensive Case–Control Study of Protective and Risk Factors for Buruli Ulcer, Southeastern Australia

Appendix

Additional Methods

Variable Creation for Analysis·

We presented details of the items, response categories, and collapsed categories for analysis (Appendix Table 3). In brief, responses to questions with frequency scales (e.g.,· never, sometimes, usually, always) were collapsed into binary categories in most instances to ensure sufficient numbers within each category for analysis. A hierarchical variable indicating the timeliness of tending to wounds was devised: category 1, persons usually/always tending cuts/scratches immediately; category 2, persons who usually/always tended cuts/scratches eventually; category 3 (reference category), all other responses, including persons leaving cuts/scratches to heal naturally.

Statistical Methods

Two participant samples were examined to explore effects of the higher proportion of holiday homeowners among cases than controls: full participant sample (comprising permanent resident and holiday homeowners) and permanent residents only. Percentages of missing data were low for most variables; if the percentage of missing data was >10%, a separate category for data missing exposure information was included in the model unless otherwise stated. Greater percentages of missing data per line item were observed for variables when participants were asked to select a frequency response (never, sometimes, usually, always) for each row of the table (e.g., whether they treated wounds, immediately, eventually, left them to heal naturally, or

other response), which might have been caused by a misunderstanding by some participants that only the line response most appropriate to them required a response.

Exploratory Factor Analysis for Potentially Protective Behaviors

Clustering of potentially protective behaviors with underlying protective factors was performed by using exploratory factor analysis. Analysis was performed by using the factormat command in Stata 16 (StataCorp LLC, https://www.stata.com), calibrating to the mean and SD matrices of the included variables, and rotating factor loadings obtained using the promax (oblique) rotation to define correlations between the derived factors. Absolute rotated factor loadings >0.3 were retained. Eigenvalues (screeplot) and the Akaike information criterion for the potential models were considered in the selection of the number of (underlying) factors retained in final factor structure; the 2-factor model was selected according to those criteria and used as a model to explain most of the variance between variables; the model had a structure that made conceptual sense.

The relationships between potentially protective health behaviors and BU case status were examined in 3 ways: individual behaviors compared with their respective reference category; categorical variables measuring the number of individual behaviors from all potentially protective behaviors and identified protective behaviors (those with odds ratio indicating a protective association) to assess effects of multiple behaviors; and as odds of BU per single unit increase in continuous factor scores for the 2 derived factors (underlying protective concepts).·Age- and sex-adjusted odds of BU for each of the potential risk or protective factors were obtained.

Sensitivity Analysis

A post-hoc sensitivity analysis was conducted to explore the robustness of the observed relationship between BCG vaccination and BU case status, given the novelty and potential significance of this finding and the number of participants reporting they were unsure if they had received the vaccine. This analysis explored the relationship between BCG vaccination and BU in age-restricted participant samples; participants were restricted to those 47–70 years of age who would have been eligible for BCG vaccination provided as part of the routine vaccination schedule for school children in Victoria from the 1950s to 1985 (*22* in main text). Participants reporting receipt of BCG vaccination and those who were unsure were classified in a single

category (under the assumption of likely vaccination through routine vaccination) and compared with age-matched participants who reported they did not receive the vaccination.

Appendix Table 1. List of postcodes included in the study areas and designated risk category at the time of the study*

Endemic areas	Suburb names	Risk category
South Eastern Bayside		
3186	Brighton, Victoria	Low
3190	Highett	Low
3192	Highett, Cheltenham (Victoria), Beaumaris (Victoria)	Low
3193	Black Rock (Victoria), Beaumaris (Victoria)	Medium
3195	Parkdale, Mordialloc, Braeside, Waterways, Aspendale Gardens, Aspendale	Low
3196	Edithvale, Bonbeach, Chelsea, Chelsea Heights	Low
**3191**	Sandringham (Victoria)	Low
**3194**	Mentone, Moorabbin Airport	Low
Frankston		
3198	Seaford (Victoria)	Medium
3199	Frankston, Frankston South	Medium
3910	Langwarrin	Low
Mornington Peninsula		
3930	Mount Eliza	Low
3931	Mornington (Victoria)	Low
3934	Mount Martha	Low
3936	Dromana, Safety Beach (Victoria), Arthurs Seat	Low
3938	McCrae	Low
3939	Rosebud, Boneo, Cape Schanck, Fingal (Victoria)	High
3940	Capel Sound	High
3941	Rye, Tootgarook, St Andrews Beach	High
3942	Blairgowrie	High
3943	Sorrento (Victoria)	High
3944	Portsea	Low
Bellarine Peninsula		
**3216**	Highton, Belmont (Victoria), Wandana Heights, Grovedale, Waurn Ponds, Marshall	Low
3222	Clifton Springs, Drysdale, Wallington, Curlewis (Victoria), Mannerim, Marcus Hill	Low
3223	Indented Head, St Leonards (Victoria), Portarlington, Bellarine	Low
3226	Ocean Grove	Medium
3227	Connewarre, Barwon Heads, Breamlea, Connewarre, Breamlea	Medium
3225	Point Lonsdale, Queenscliff (Victoria), Point Lonsdale, Swan Bay (Victoria), Swan Island, Swan Bay (Victoria)	Medium
Aireys Inlet and surrounds		
**3230**	Anglesea	Low
3231	Eastern View, Fairhaven, Aireys Inlet, Moggs Creek, Big Hill (Surf Coast, Victoria)	Low

*Postcodes in bold text were not included in the participant study sample.

Appendix Table 2. Variables, questionnaire items, and response and collapsed categories used for analyses*

Displayed variable	Questionnaire item	Response categories	Collapsed categories
Employment status	What is your employment status? (tick as many boxes as fits)	Employed; student; home duties; retired; unemployed	1, Employed; 2, Unpaid employment (student, home duties, unemployed if not also employed); 3, Retired
Occupation exposure risk (for those working in affected areas only)	What is your employment status? If employed, do you work from home? If "No," is your job based in the affected area? What proportion of your time do you spend outside as part of your occupation? Are you in contact with the soil during your work?	As above: no, yes; no, yes; time outside: none, <1/4, 1/4 −3/4, >3/4; no, yes, sometimes	If employed and working at home for permanent residents or jobs based in the affected area: Indoor: proportion spent outside = none; Outdoors, without soil contact: proportion outdoors >1/4 and no soil contact; Outdoor with soil contact: proportion outdoors >1/4 and yes soil contact (yes/sometimes)
Skin injuries at work (those working in affected areas only)	Do you ever get injuries to the skin on your limbs at work?	no, yes, sometimes	If employed and based in affected areas, no, yes (yes/sometimes)
Long sleeves and pants (those working outdoors in affected areas)	Do you wear long sleeved shirts and long pants when you work?	no, yes, sometimes	If employed and based in affected areas, no, yes (yes/sometimes)
Gardening	How often do your garden?	Daily, weekly, monthly, rarely, I do not garden	No, don't garden/rarely; Yes, garden (daily, weekly, monthly)
Gardening frequency	How often do your garden?	Daily, weekly, monthly, rarely, I do not garden	Rarely/I do not garden; monthly, weekly, daily
Gardening injury frequency	When you garden, do you injure yourself (e·g, with thorns)?	Frequently, occasionally, never	Frequently, occasionally, never
Outdoor activities	Please estimate the number of days you engage in any of these activities when you are in the affected area during each 6-month period. If you do not do the activity, please leave the row blank.	NA	NA
Beach walks/jogging	Please estimate the number of days you engage in any of these activities when you are in the affected area during each 6-month period. If you do not do the activity, please leave the row blank.	Number of days (Sep–Feb, out of 181 d); Number of days (Mar–Aug, out of 184 d)	No, zero days/blank response; Yes, any days reported
Wetland walks/jogging	Please estimate the number of days you engage in any of these activities when you are in the affected area during each 6-month period. If you do not do the activity, please leave the row blank.	Number of days (Sep–Feb, out of 181 d); Number of days (Mar–Aug, out of 184 d)	No, zero days/blank response; Yes, any days reported
Bushwalking	Please estimate the number of days you engage in any of these activities when you are in the affected area during each 6-month period. If you do not do the activity, please leave the row blank.	Number of days (Sep–Feb, out of 181 d); Number of days (Mar–Aug, out of 184 d)	No, zero days/blank response; Yes, any days reported
Golf	Please estimate the number of days you engage in any of these activities when you are in the affected area during each 6-month period. If you do not do the activity, please leave the row blank.	Number of days (Sep–Feb, out of 181 d); Number of days (Mar–Aug, out of 184 d)	No, zero days/blank response; Yes, any days reported
Sports on an oval (e.g., AFL, soccer, rugby)	Please estimate the number of days you engage in any of these activities when you are in the affected area during each 6-month period. If you do not do the activity, please leave the row blank.	Number of days (Sep–Feb, out of 181 d); Number of days (Mar–Aug, out of 184 d)	No, zero days/blank response; Yes, any days reported
Swimming in local lakes/rivers	Please estimate the number of days you engage in any of these activities when you are in the affected area during each 6-month period. If you do not do the activity, please leave the row blank.	Number of days (Sep–Feb, out of 181 d); Number of days (Mar–Aug, out of 184 d)	No, zero days/blank response; Yes, any days reported

Displayed variable	Questionnaire item	Response categories	Collapsed categories
Sailing	Please estimate the number of days you engage in any of these activities when you are in the affected area during each 6-month period. If you do not do the activity, please leave the row blank.	Number of days (Sep–Feb, out of 181 d); Number of days (Mar–Aug, out of 184 d)	No, zero days/blank response; Yes, any days reported
Outdoor barbeques	Please estimate the number of days you engage in any of these activities when you are in the affected area during each 6-month period. If you do not do the activity, please leave the row blank.	Number of days (Sep–Feb, out of 181 d); Number of days (Mar–Aug, out of 184 d)	No, zero days/blank response; Yes, any days reported
Other activities (please state)	Please estimate the number of days you engage in any of these activities when you are in the affected area during each 6-month period. If you do not do the activity, please leave the row blank.	Number of days (Sep–Feb, out of 181 d); Number of days (Mar–Aug, out of 184 d)	No, zero days/blank response; Yes, any days reported
Any reported outdoor activities	Derived count	NA	No, no outdoor activities; Yes, any of the above or other outdoor activities reported
Days of outdoor activities in warmer months	Please estimate the number of days you engage in any of these activities when you are in the affected area during each 6-month period. If you do not do the activity, please leave the row blank.	Number of days (Sep–Feb, out of 181 d)	Addition of all reported days for warmer months (Sep–Feb): 1, lowest tertile (including none); 2; 3, highest tertile
Days of outdoor activities in cooler months	Please estimate the number of days you engage in any of these activities when you are in the affected area during each 6-month period. If you do not do the activity, please leave the row blank.	Number of days (Mar–Aug, out of 184 d)	Addition of all reported days for cooler months (Mar–Aug): 1, lowest tertile (including none); 2; 3, highest tertile
Wildlife seen on or around property in affected area	Do you see wild or feral mammals (e.g., possums, koalas, fruit bats, bandicoots, foxes, rodents) on and around your property or holiday accommodation in the affected area?	Yes, what species?; no	Bats (no, yes); foxes (no, yes); rabbits (no, yes);
Rodents (exotic or native or reported rodent activity related to pest control)	Do you see wild or feral mammals (e.g., possums, koalas, fruit bats, bandicoots, foxes, rodents) on and around your property or holiday accommodation in the affected area? What kind of pests are controlled at your property? Do you see signs of rodent activity around your property? (e.g., feces, nibbled containers)	Yes, what species? No. Pests: insects, rodents, possums, birds, other (please state); no, yes	Yes, exotic or native rodents observed, rodent pests, or yes to rodent activity
Possums, possum species	If you aware of possums on your property/holiday accommodation, do you know what kind they are?	Not sure, ringtail, brushtail, NA	Possums, yes, or possums identified in wildlife question
If possums, frequency of presence	If possums are on your property, how often are they present?	Frequently/always; occasionally	Never/occasionally; frequently/always
Number of possums present	Do you know how many possums are present?	1–2, 3–5, >5, not sure	1–2, 3–5, >5, not sure
Possum feces in surroundings of property	Do you find possum feces in the surroundings of your property or holiday accommodation in the affected area?	No; yes, but only small amounts; yes, large amounts; unsure	No; yes, but only small amounts; yes, large amounts; unsure
Pets: dog, cat, bird, other	Do you have any pets(s)? If you answered yes to question 16a, what pet do you have?	no, yes; dog, cat, bird, other (please specify)	no, yes
Wounded by pet	If you answered yes to question 16a, do you get bitten, scratched, or injured by your pet?	Frequently, occasionally, never	Never/no pet, occasionally/frequently
Pet has fleas	If you answered yes to question (pets), does your pet ever have fleas?	no, yes	no/no pet, yes
Regularity of contact with livestock	How regular is your contact with livestock in the affected area (including horses)?	Frequent (>1×/mo), occasional (<1×/mo), never	Never, occasional/frequent

Displayed variable	Questionnaire item	Response categories	Collapsed categories
Drinking	What sort of water do you use for the following purpose (please check which applies)? Drinking: town water, unfiltered; town water, filtered; tank rain water, unfiltered; tank rain water, filtered; bore water; bottled water	Always, usually, sometimes, never	No (never); yes (sometimes/usually/always)
Skin Contact	What sort of water do you use for the following purpose (please check which applies)? Bathing/showering: town water, tank rain water, bore water; Gardening: town water, tank rain water, bore water	Always, usually, sometimes, never	Combining bathing/showering and gardening responses: no (never); yes (sometimes/usually/always)
Bird bath	Do you have a birdbath in your property in the affected area?	no, yes	no, yes
Other water sources	Do you have another type of water feature on your property in the affected area (e.g., sculpture, bowl, swimming pool, etc.)?	no, yes (please specify)	None (no), bowl/dish/drain/pot/other, pond, water feature, pool, water tank/various
Pond at the property	Do you have another type of water feature in your property in the affected area (e.g., sculpture, bowl, swimming pool, etc.)?	Pond specified	no, yes (pond specified)
Potting mix, fertilizer	Do you use any of these products?	yes, no; brand name, how often per year, where purchased	No, yes
Top soil or mulch (previous 12 mo)	In the past 12 months, have you had topsoil or mulch delivered or purchased for your garden in the affected area?	No, yes (if yes, topsoil, mulch?)	No, yes
Major renovations (previous 12 mo)	Have you had any major renovations (involving earthworks and landscaping) on your property in the affected area in the past 12 mo?	No, yes (describe), unknown	No, yes
Earthworks (previous 12 mo)	Have there been any earthworks or major renovations in the immediate area outside your home in the affected area in the past 12 mo?	No, yes (describe), unknown	No, yes, unknown
Sewerage	How is sewage disposed of at your property?	Main sewerage system, septic tank, other, unknown	Main sewerage system, septic tank (includes those with septic and mains sewerage), other (other/unknown)
Sewerage works (previous 12 mo)	Have you had sewerage works on your house or near your house (i·e· in the same street/neighboring street) in the last 12 mo?	No, yes, unknown	No, yes, unknown
Frequent presence at residence/holiday home: mosquitoes, March flies, sand flies (midges), other biting insects	Are these biting insects frequently seen around your home or holiday residence in the affected area? (Please tick as many as applicable)·Picture and adults size description to help with identification.	Check box, please specify for other	No, yes (if checked)
Frequency of being bitten: mosquitoes, March flies, sand flies (midges), other insects	How often do you get bitten by mosquitoes? How often do you get bitten by March flies? How often do you get bitten by sand flies (midges)? How often do you get bitten by other insects?	Frequently, occasionally, never	Never, occasionally/frequently
Tendency to scratch insect bites	Do you tend to scratch your insect bites?	I never get bitten, no, yes	No/never get bitten, yes
Any pest control	How often do you have to control for pests at your property in the affected area?	Frequently (>1×/y), occasionally (<1×/y), never	Frequent, occasional, never
Pest control: insect, possum, rodent	What kind of pests are controlled at your property?	Check box, please specify for other	no, yes
Covers preexisting wounds with dressing	If you have a preexisting cut or scratch or other wound when you go out to garden, are working, or	Always, usually, sometimes, never (for each row)	Response to question (a) always/usually; never/sometimes/missing

Displayed variable	Questionnaire item	Response categories	Collapsed categories
	take part in other outdoor activities in the affected area, do you generally: (please tick a response for each row): (a) ensure the area is covered with a dressing; (b) leave it open to the air; (c) other (please specify)		
Timeliness of tending to cuts and scratches from outdoors (hierarchical derived variable)	If you cut or scratch yourself during gardening, working outside, or outdoor activities, do you generally·(please tick a response for each row): (a) immediately stop what you are doing and wash the area, then apply antiseptic or dressings (bandaids, etc.) to the area; (b) eventually clean and apply antiseptic or dressings to the area when activity is completed; (c) leave the area to heal naturally (i.e., do not apply dressings or antiseptic); (d) other (please specify)?	Always, usually, sometimes, never (for each row)	Immediately (wash and dressing or antiseptic), if usually/always for question (a); eventually (wash and dressing or antiseptic), if not immediately and usually/always for question (b); leaves to heal naturally or other response, if not either immediately or eventually, or any other response, including missing·
Tending to cuts/scratches immediately (binary)	If you cut or scratch yourself during gardening, working outside, or outdoor activities, do you generally·(please tick a response for each row): (a) immediately stop what you are doing and wash the area, then apply antiseptic or dressings (bandaids, etc.) to the area?·	Always, usually, sometimes, never (for each row)	Response to question (a): always/usually; never/sometimes/missing
Washes hands after outdoor activity; showers after outdoor activity	After gardening or working outdoors do you: (a) shower immediately,(b) wash your hands, (c) other (please specify)?	Always, usually, sometimes, never (for each row, but no specific instruction to complete each row)	Never/sometimes, usually/always
Clothing covering arms and legs	When you are gardening or involved in outdoor activities, do you usually: (a) cover your arms (i.e., you wear long sleeved t-shirts, etc.), (b) cover your legs (i.e., you wear long pants, etc.)?	Always, usually, sometimes, never (for each row, but no specific instruction to complete each row)	Never/sometimes/seasonally; usually/always for (a) or (b), either arms or legs; usually/always for (a) and (b), both arms and legs
Gardening gloves	Do you wear gloves when you garden?	Always, usually, sometimes, never	Never, sometimes/usually/always
Shoes other than thongs (warmer months)	Do you wear open shoes (e.g.,· thongs, sandals) outside during the following months when you are in the affected area (please tick the appropriate boxes to indicate how often you wear them)? Warmer months (Sep–Feb)	Always, usually, sometimes, never	Never/sometimes (for those responding that they usually/always wear open shoes); usually/always (for those responding that they never or sometimes wear open shoes)
Multiple examined behaviors	Derived count	Derived from counts of all potentially protective behaviors examined: 1, covers preexisting wounds; 2, tends to cuts and scratches immediately; 3, washes hands; 4, showers after outdoor activity; 5, insect repellent use in warm months; 6, clothing coverage; 7, gardening gloves; 8, closed shoes outside in warm months	0–1, 2–3, 4–5, ≥6
Multiple behaviors identified as protective	Derived count	1, tends to cuts and scratches immediately; 2, clothing coverage of arms and legs; 3,·insect repellent use in warm months	None, 1, 2, 3

*AFL, Australian football league; NA, not applicable.

Appendix Table 3. Medical history of patients and controls (all participants, residents only) and associations with Buruli ulcer*

Medical history	All participants			Residents only		
	Controls	Cases	aOR† (95% CI)	Controls	Cases	aOR† (95% CI)
No. participants	481	245	NA	469	171	NA
Diabetes						
No	458 (96)	224 (92)	1.0	446 (96)	154 (91)	1.0
Yes	19 (4)	19 (8)	2.26 (1.13–4.49)	19 (4)	15 (9)	2.33 (1.13–4.80)
Hypothyroidism						
No	455 (95)	230 (94)	1.0	443 (95)	159 (94)	1.0
Yes	22 (5)	13 (5)	1.58 (0.76–3.31)	22 (5)	10 (6)	1.64 (0.74–3.64)
Kidney disease						
No	475 (99)	240 (98)	1.0	463 (99.6)	168 (99.4)	1.0
Yes	2 (0.4)	3 (1)	2.97 (0.48–18.34)	2 (0.4)	10 (0.6)	1.62 (0.14–18.71)
Liver cirrhosis						
No	475 (99.6)	243 (99)	NA	463 (99.6)	169 (100)	NA
Yes	2 (0.4)	0 (0)	NA	2 (0.4)	0 (0)	NA
HIV						
No	477 (100)	243 (100)	NA	465 (100)	169 (100)	NA
Yes	0 (0)	0 (0)	NA	0 (0)	0 (0)	NA
Cancer						
No	435 (91)	225 (93)	1.0	424 (91)	158 (93)	1.0
Yes	42 (9)	18 (7)	0.87 (0.48–1.57)	41 (9)	11 (7)	0.69 (0.35–1.40)
Pregnancy						
No	477 (100)	241 (99)	NA	465 (100)	167 (99)	NA
Yes	0 (0)	2 (0.8)	NA	0 (0)	2 (1)	NA
Any reported immune-compromising condition, excluding cancer and diabetes						
No	458 (95)	228 (93)	1.0	446 (95)	162 (95)	1.0
Yes	23 (5)	17 (7)	1.55 (0.80–3.01)	23 (5)	9 (5)	1.13 (0.51–2.54)
Medication: prednisolone						
No	441 (92)	181 (74)	1.0	430 (92)	122 (71)	1.0
Yes, prednisolone	17 (4)	18 (7)	2.56 (1.28–5.13)	16 (3)	13 (8)	2.71 (1.26–5.82)
Medication response missing	23 (5)	46 (19)	4.65 (2.71–7.98)	23 (5)	36 (21)	5.25 (2.80–9.23)
Tobacco-smoking habits						
Nonsmoker	431 (90)	214 (89)	1.0	421 (91)	146 (87)	1.0
Irregular	15 (3)	9 (4)	0.97 (0.40–2.34)	13 (3)	6 (4)	1.17 (0.42–3.29)
Regular	31 (7)	18 (7)	1.05 (0.57–1.98)	31 (7)	16 (10)	1.38 (0.73–2.63)
BCG tuberculosis vaccination						
No	112 (23)	70 (29)	1.0	109 (23)	48 (28)	1.0
Yes	220 (46)	75 (31)	0.59 (0.39–0.90)	215 (46)	51 (30)	0.56 (0.35–0.89)
Unsure	149 (31)	100 (41)	1.04 (0.70–1·57)	145 (31)	72 (42)	1.10 (0.70–1.72)
Timing of last BCG tuberculosis vaccination (if vaccinated)						
Within 20 y	14 (6)	6 (8)	0.96 (0.30–3.08)	14 (7)	4 (8)	1.40 (0.36–5.42)
>20 y	175 (80)	57 (76)	1.0	170 (79)	36 (71)	1.0
Missing	31 (14)	12 (16)	1.15 (0.55–2.41)	31 (14)	11 (22)	1.91 (0.84–4.34)

*Values are no. (%) except as indicated. aOR, adjusted odds ratio; BCG, Bacille Calmette-Guérin; NA, not applicable.
†Adjusted for age and sex.

Appendix Table 4. Occupational exposure-related factors according to case study and control populations (all participants, residents only) and associations with Buruli ulcer*

	All participants			Residents only		
Exposure-related factors	Controls	Cases	aOR† (95% CI)	Controls	Cases	aOR† (95% CI)
No. participants	481	245	NA	469	171	NA
Occupation exposure risk‡						
Indoor	57 (48)	11 (28)	1.0	57 (49)	10 (29)	1.0
Outdoor, no soil contact	20 (17)	9 (23)	2.11 (0.70–6.38)	19 (16)	9 (26)	2.15 (0.70–6.58)
Outdoor, with soil contact	42 (35)	20 (50)	2.89 (1.01–8.25)	41 (35)	16 (46)	2.67 (0.87–8.22)
Proportion of time spent outside as part of job‡						
None	56 (47)	11 (27)	1.0	56 (47)	10 (28)	1.0
<0.25	23 (19)	10 (24)	2.20 (0.80–6.10)	22 (19)	8 (22)	1.93 (0.62–6.02)
0.25–0.75	15 (13)	11 (27)	4.12 (1.25–13.57)	14 (12)	9 (25)	3.56 (1.03–6.02)
>0.75	26 (22)	9 (22)	1.63 (0.59–4.50)	26 (22)	9 (25)	2.03 (0.57–7.28)
Skin injuries at work‡						
No	55 (48)	17 (43)	1.0	54 (48)	15 (43)	1.0
Yes/Sometimes	60 (52)	23 (58)	1.03 (0.45–2.37)	59 (52)	20 (57)	1.03 (0.42, 2.49)
Long sleeves and pants§						
No	18 (29)	10 (34)	1.0	17 (28)	9 (36)	1.0
Yes/Sometimes	44 (71)	19 (66)	0.85 (0.31–2.34)	43 (72)	16 (64)	0.85 (0.30–2.43)

*Values are no. (%) except as indicated. aOR, adjusted odds ratio, NA, not applicable.
†Adjusted for age and sex.
‡For persons working in affected areas only.
§For persons working outdoors in affected areas.

Appendix Table 5. Animal exposures (wildlife, pets, other animals) according to case study and control populations and associations with Buruli ulcer*

	All participants			Residents only		
Animal exposures†	Controls	Cases	aOR‡ (95% CI)	Controls	Cases	aOR‡ (95% CI)
No. participants	481	245	NA	469	171	NA
Bats						
No	434 (92)	226 (92)	1.0	424 (92)	155 (91)	1.0
Yes	41 (9)	19 (8)	0.81 (0.44–1.47)	39 (8)	16 (9)	0.97 (0.51–1.83)
Foxes						
No	267 (56)	153 (62)	1.0	259 (56)	100 (58)	1.0
Yes	208 (44)	92 (38)	0.66 (0.47–0.92)	204 (44)	71 (42)	0.83 (0.56–1.22)
Rodents, exotic or native or reported rodent activity related to pest control						
No	166 (35)	97 (40)	1.0	161 (35)	61 (36)	1.0
Yes	311 (65)	148 (60)	0.78 (0.56–1.08)	304 (65)	110 (64)	0.98 (0.67–1.43)
Rabbits						
No	452 (95)	241 (98)	1.0	441 (95)	167 (98)	1.0
Yes	23 (5)	4 (2)	0.30 (0.10–0.91)	22 (5)	4 (2)	0.45 (0.15–1.40)
Possums						
No	51 (11)	11 (4)	1.0	49 (11)	4 (2)	1.0
Yes	425 (89)	234 (96)	2.33 (1.15-4.71)	415 (89)	167 (98)	5.30 (1.82–15.49)
If possums, brushtail						
No	266 (63)	147 (64)	1.0	259 (63)	102 (62)	1.0
Yes	155 (37)	84 (36)	0.92 (0.65–1.30)	152 (37)	63 (38)	0.92 (0.63–1.37)
If possums, ringtail						
No	150 (36)	80 (35)	1.0	145 (35)	51 (31)	1.0
Yes	271 (64)	151 (65)	1.03 (0.73–1.46)	266 (65)	114 (69)	1.20 (0.81–1.79)
If possums, unsure of type	124 (29)	66 (28)	NA	120 (29)	41 (25)	NA
If possums, frequency of presence						
Never/occasionally	116 (28)	57 (25)	1.0	113 (28)	44 (27)	1.0
Frequently/always	302 (72)	173 (75)	1.04 (0.72–1.51)	295 (72)	121 (73)	1.05 (0.69–1.58)
No. possums present						
0	47 (10)	10 (4)	1.0	45 (10)	4 (2)	1.0
1–2	120 (25)	54 (22)	1.95 (0.89–4.27)	117 (25)	44 (26)	4.52 (1.48–13.81)
3–5	82 (17)	59 (24)	2.93 (1.31–6.53)	80 (17)	38 (22)	5.51 (1.76–17.23)
>5	51 (11)	36 (15)	3.07 (1.30–7.21)	51 (11)	24 (14)	6.06 (1.85–19.83)
Not sure	176 (37)	85 (35)	2.20 (1.02–4.76)	171 (37)	60 (35)	4.54 (1.50–13.79)
Possum feces in surroundings of property						
No	95 (20)	28 (12)	1.0	93 (20)	22 (13)	1.0
Yes,small amounts	179 (38)	84 (35)	1.44 (0.85–2.42)	174 (38)	59 (35)	1.41 (0.79–2.51)
Yes, large amounts	143 (30)	95 (40)	1.97 (1.16–3.34)	139 (30)	65 (38)	1.88 (1.05–3.36)
Unsure	57 (12)	33 (14)	1.78 (0.95–3.34)	56 (12)	23 (14)	1.66 (0.83–3.32)
Feeding birds grain, seed, etc. in garden						
No	309 (64)	174 (71)	1.0	299 (64)	115 (67)	1.0

	All participants			Residents only		
Animal exposures†	Controls	Cases	aOR‡ (95% CI)	Controls	Cases	aOR‡ (95% CI)
Yes/sometimes	171 (36)	71 (29)	0.75 (0.53–1.07)	169 (36)	56 (33)	0.86 (0.59–1.27)
Pet bird						
No	444 (93)	237 (97)	1.0	432 (93)	164 (96)	1.0
Yes	33 (7)	8 (3)	0.45 (0.20–1.01)	33 (7)	7 (4)	0.54 (0.23–1.29)
Pet cat						
No	409 (86)	208 (85)	1.0	398 (85)	141 (82)	1.0
Yes	69 (14)	37 (15)	0.99 (0.63–1.56)	68 (15)	30 (18)	1.10 (0.68–1.80)
Pet dog						
No	272 (57)	128 (52)	1.0	264 (57)	91 (53)	1.0
Yes	207 (43)	117 (48)	1.15 (0.83–1.60)	203 (43)	80 (47)	1.11 (0.77–1.61)
Pet, other						
No	448 (93)	236 (96)	1.0	437 (93)	163 (95)	1.0
Yes	33 (7)	9 (4)	0.45 (0.20–0.98)	32 (7)	8 (5)	0.60 (0.27–1.37)
Wounded by pets						
Never/no pet	378 (79)	217 (89)	1.0	367 (79)	146 (85)	1.0
Occasional/frequent	99 (21)	28 (11)	0.42 (0.28–0.72)	98 (21)	25 (15)	0.57 (0.35–0.93)
Pet has fleas						
No/no pet	396 (86)	206 (87)	1.0	384 (86)	138 (84)	1.0
Yes	62 (14)	31 (13)	0.96 (0.59–1.55)	62 (14)	27 (16)	1.16 (0.70–1.94)
Regularity of contact with livestock						
Never	392 (91)	212 (91)	1.0	385 (91)	144 (88)	1.0
Occasional/frequent	41 (9)	20 (9)	0.88 (0.48–1.62)	38 (9)	19 (12)	1.41 (0.75–2.66)
Tea trees§						
No	154 (33)	51 (21)	1.0	151 (33)	40 (24)	1.0
Yes	312 (67)	189 (79)	1.59 (1.07–2.37)	303 (67)	129 (76)	1.72 (1.10–2.69)

*Values are no. (%) except as indicated. aOR, adjusted odds ratio; NA, not applicable.
†Wildlife seen on or around property in affected areas.
‡Adjusted for age and sex.
§Common habitat for possums.

Appendix Table 6. Water usage and environmental water sources according to case study and control populations and associations with Buruli ulcer*

	All participants			Residents only		
Water use and sources	Controls	Cases	aOR† (95% CI)	Controls	Cases	aOR† (95% CI)
No. participants	481	245	NA	469	171	NA
Drinking unfiltered town water						
No	124 (26)	40 (16)	1.0	118 (25)	27 (16)	1.0
Yes	354 (74)	204 (84)	1.57 (1.05–2.36)	348 (75)	143 (84)	1.65 (1.03–2.63)
Drinking filtered town water						
No	280 (59)	167 (68)	1.0	277 (60)	114 (67)	1.0
Yes	197 (41)	77 (32)	0.64 (0.46–0.90)	188 (40)	56 (33)	0.74 (0.51–1.07)
Drinking bore water						
No	18 (4)	18 (7)	1.0	461 (99)	169 (99)	1.0
Yes	463 (96)	226 (93)	0.20 (0.02–1.74)	5 (1)	1 (1)	0.34 (0.04–3.04)
Drinking bottled water						
No	319 (67)	152 (62)	1.0	311 (67)	104 (61)	1.0
Yes	159 (33)	92 (38)	1.08 (0.77–1.52)	155 (33)	66 (39)	1.16 (0.79–1.71)
Skin contact with town water, bathing/gardening						
No	1 (0.2)	1 (0.4)	1.0	1 (0.2)	1 (1)	1.0
Yes	475 (99.8)	242 (99.6)	0.49 (0.03–7.85)	463 (99.8)	168 (99)	0.33 (0.02–5.43)
Skin contact with tank water, bathing/gardening						
No	321 (67)	181 (74)	1.0	311 (67)	120 (71)	1.0
Yes	155 (33)	62 (26)	0.77 (0.54–1.10)	153 (33)	49 (29)	0.83 (0.56–1.23)
Skin contact with gray water, bathing/gardening						
No	439 (93)	228 (95)	1.0	429 (93)	158 (93)	1.0
Yes	35 (7)	13 (6)	0.79 (0.40–1.55)	33 (7)	11 (7)	0.97 (0.47–2.00)
Skin contact with bore water, bathing/gardening						
No	392 (82)	181 (74)	1.0	385 (83)	129 (76)	1.0
Yes	84 (18)	62 (26)	1.34 (0.90–2.02)	79 (17)	40 (24)	1.56 (0.98–2.50)
Bird bath						
No	224 (47)	127 (52)	1.0	217 (47)	76 (44)	1.0
Yes	252 (53)	117 (48)	0.95 (0.68–1.33)	247 (53)	95 (56)	1.30 (0.88–1.90)
Other water sources						
None	254 (55)	135 (57)	1.0	247 (55)	87 (53)	1.0
Bowl/dish/drain/pot/other	61 (13)	28 (12)	0.90 (0.54–1.49)	60 (13)	19 (12)	0.89 (0.50–1.60)
Pond	45 (10)	30 (13)	1.34 (0.79–2.26)	44 (10)	26 (16)	1.59 (0.91–2.79)

Water use and sources	All participants Controls	Cases	aOR† (95% CI)	Residents only Controls	Cases	aOR† (95% CI)
No. participants	481	245	NA	469	171	NA
Water feature	31 (7)	11 (5)	0.74 (0.35–1.56)	31 (7)	7 (4)	0.67 (0.27–1.62)
Pool	41 (9)	19 (8)	0.66 (0.36–1.22)	39 (9)	11 (7)	0.65 (0.31–1.35)
Water tank/various	27 (6)	15 (6)	1.02 (0.52–2.01)	27 (6)	14 (9)	1.31 (0.65–2.64)
Pond at the property						
No	436 (91)	215 (88)	1.0	425 (91)	145 (85)	1.0
Yes	45 (9)	30 (12)	1.46 (0.88–2.42)	44 (9)	26 (15)	1.69 (0.99–2.89)

*Values are no. (%) except as indicated. aOR, adjusted odds ratio, NA, not applicable.
†Adjusted for age and sex.

Appendix Table 7. Environmental sources related to soil and sewerage according to case study and control populations and associations with Buruli ulcer*

Exposure sources	All participants Controls	Cases	a OR† (95% CI)	Residents only Controls	Cases	aOR† (95% CI)
No. participants	481	245	NA	469	171	NA
Potting Mix						
No	98 (21)	80 (34)	1.0	93 (20)	36 (22)	1.0
Yes	370 (79)	156 (66)	0.56 (0.39–0.82)	363 (80)	130 (78)	0.97 (0.62–1.54)
Fertilizer						
No	132 (29)	78 (35)	1.0	128 (29)	42 (27)	1.0
Yes	323 (71)	142 (65)	0.76 (0.53–1.10)	315 (71)	112 (73)	1.08 (0.70–1.66)
Top soil, last 12 mo						
No	405 (86)	207 (86)	1.0	398 (86)	143 (85)	1.0
Yes	68 (14)	34 (14)	0.88 (0.56–1.39)	64 (14)	25 (15)	1.04 (0.63–1.74)
Mulch, last 12 mo						
No	295 (63)	163 (68)	1.0	288 (63)	117 (70)	1.0
Yes	176 (37)	76 (32)	0.75 (0.53–1.06)	172 (37)	50 (30)	0.72 (0.48–1.07)
Major renovations, last 12 mo						
No	392 (83)	200 (84)	1.0	381 (83)	142 (86)	1.0
Yes	80 (17)	39 (16)	0.86 (0.55–1.32)	79 (17)	24 (14)	0.80 (0.48–1.33)
Earthworks, last 12 mo						
No	281 (61)	113 (68)	1.0	281 (61)	113 (68)	1.0
Yes	144 (31)	42 (25)	0.71 (0.49–1.03)	144 (31)	42 (25)	0.74 (0.49–1.13)
Unknown	36 (8)	12 (7)	1.21 (0.70–2.09)	36 (8)	12 (7)	0.83 (0.41–1.68)
Sewerage						
Main sewerage system	354 (75)	163 (67)	1.0	343 (74)	128 (77)	1.0
Septic tank	110 (23)	71 (29)	1.24 (0.80–1.91)	109 (24)	37 (22)	1.05 (0.62–1.79)
Other	11 (2)	8 (3)	1.20 (0.45–3.18)	11 (2)	3 (2)	0.70 (0.19–2.68)
Sewerage works, last 12 mo						
No	275 (58)	133 (55)	1.0	269 (58)	93 (56)	1.0
Yes	131 (28)	60 (25)	0.84 (0.56–1.26)	125 (27)	43 (26)	1.07 (0.68–1.68)
Unknown	68 (14)	48 (20)	1.43 (0.91–2.24)	68 (15)	31 (19)	1.32 (0.79–2.20)

*Values are no. (%) except as indicated. aOR, adjusted odds ratio, NA, not applicable.
†Adjusted for age and sex.

Appendix Table 8. Insect exposure and pest control according to case study and control populations and associations with Buruli ulcer*

	All participants				Residents only			
Characteristics	Controls	Cases	aOR† (95%CI)	aOR‡ (95% CI)	Controls	Cases	aOR† (95%CI)	aOR‡ (95% CI)
No. participants	481	245	NA	NA	469	171	NA	NA
Frequent presence, residence/holiday home								
Mosquitoes								
No	23 (5)	12 (5)	1.0	NA	23 (5)	9 (5)	1.0	NA
Yes	454 (95)	233 (95)	0.87 (0.41–1.84)	NA	442 (95)	162 (95)	0.91 (0.40–2.06)	NA
March flies								
No	199 (42)	106 (44)	1.0	NA	198 (43)	75 (44)	1.0	NA
Yes	277 (58)	137 (56)	0.79 (0.56–1.12)	NA	266 (57)	95 (56)	0.98 (0.66–1.45)	NA
Sand flies								
No	357 (75)	167 (69)	1.0	NA	351 (76)	122 (73)	1.0	NA
Yes	116 (25)	74 (31)	1.29 (0.90–1.85)	NA	110 (24)	46 (27)	1.17 (0.77–1.78)	NA
Other biting insects								
No	448 (95)	227 (93)	1.0	NA	436 (94)	158 (92)	1.0	NA
Yes	26 (5)	18 (7)	1.63 (0.85–3.11)	NA	26 (6)	13 (8)	1.36 (0.66–2.78)	NA
Frequency of being bitten								
Mosquitoes								
Never	30 (6)	25 (10)	1.0	1.0	28 (6)	18 (11)	1.0	1.0
Occasionally, frequently	450 (94)	220 (90)	0.54 (0.30–0.96)	0.59 (0.33–1.06)	440 (94)	153 (89)	0.49 (0.26–0.93)	0.56 (0.29–1.07)
March Flies								
Never	188 (39)	96 (40)	1.0	1.0	184 (40)	70 (41)	1.0	1.0
Occasionally, frequently	289 (61)	146 (60)	0.82 (0.57–1.18)	0.86 (0.59–1.24)	281 (60)	99 (59)	0.98 (0.65–1.49)	1.07 (0.70–1.64)
Sand Flies/ Midges								
Never	290 (61)	164 (67)	1.0	1.0	283 (61)	119 (70)	1.0	1.0
Occasionally, frequently	184 (39)	79 (33)	0.73 (0.52–1.02)	0.76 (0.54–1.07)	179 (39)	51 (30)	0.67 (0.46–0.997)	0.72 (0.49–1.08)
Other biting insects								
Never	257 (54)	148 (61)	1.0	1.0	249 (54)	104 (62)	1.0	1.0
Occasionally, frequently	217 (46)	93 (39)	0.73 (0.53–1.02)	0.76 (0.54–1.05)	213 (46)	64 (38)	0.70 (0.48–1.02)	0.73 (0.50–1.06)
Tendency to scratch insect bites								
No, or never get bitten	175 (37)	102 (42)	1.0	NA	173 (37)	73 (43)	1.0	NA
Yes	303 (63)	141 (58)	0.74 (0.53–1.03)	NA	293 (63)	97 (57)	0.74 (0.51–1.07)	NA
Pest Control								
Any Pest Control								
Never	177 (37)	95 (40)	1.0	NA	173 (37)	66 (39)	1.0	NA
Occasional	229 (48)	117(49)	0.94 (0.66–1.33)	NA	224 (48)	87 (51)	1.03 (0.70–1.52)	NA
Frequent	69 (15)	28 (12)	0.71 (0.42–1.18)	NA	66 (14)	16 (9)	0.60 (0.32–1.12)	NA
Insect control								
No	341 (72)	166 (70)	1.0	NA	335 (73)	121 (72)	1.0	NA
Yes	130 (28)	72 (30)	1.14 (0.80–1.62)	NA	124 (27)	46 (28)	1.02 (0.68–1.53)	NA
Possum control								
No	425 (90)	223 (94)		NA	414 (90)	157 (94)	1.0	NA
Yes	46 (10)	15 (6)	0.63 (0.34–1.17)	NA	45 (10)	10 (6)	0.59 (0.29–1.21)	NA
Rodent control								
No	272 (58)	141 (59)	1.0	NA	263 (57)	91 (54)	1.0	NA
Yes	200 (42)	97 (41)	0.87 (0.62–1.21)	NA	197 (43)	76 (46)	1.12 (0.77–1.62)	NA

*Values are no. (%) except as indicated. aOR, adjusted odds ratio, NA, not applicable.
†Adjusted for age and sex.
‡Adjusted for age, sex, and insect repellent use.

Appendix Table 9. Gardening exposures and other outdoor activities according to case study and control populations and associations with Buruli ulcer*

	All participants			Residents only		
Exposures	Controls	Cases	aOR† (95% CI)	Controls	Cases	aOR† (95% CI)
No. participants	481	245	NA	469	171	NA
Gardening						
No, don't garden/rarely garden	81 (17)	69 (28)	1.0	76 (16)	34 (20)	1.0
Yes, garden	393 (83)	174 (72)	0.50 (0.34–0.74)	386 (84)	135 (80)	0.74 (0.46–1.18)
Gardening frequency						
Rarely/I do not garden	81 (17)	69 (28)	1.0	76 (16)	34 (20)	1.0
Monthly	86 (18)	39 (16)	0.51 (0.31–0.86)	84 (18)	26 (15)	0.64 (0.35–1.18)
Weekly	204 (43)	86 (35)	0.48 (0.31–0.74)	201 (44)	68 (40)	0.73 (0.44–1.21)
Daily	103 (22)	49 (20)	0.55 (0.33–0.91)	101 (22)	41 (24)	0.87 (0.49–1.55)
Gardening injury frequency						
Never	93 (20)	59 (25)	1.0	90 (20)	36 (22)	1.0
Occasionally	322 (68)	154 (65)	0.82 (0.55–1.22)	313 (68)	109 (66)	0.96 (0.61–1.52)
Frequently	57 (12)	24 (10)	0.72 (0.39–1.30)	57 (12)	21 (13)	0.95 (0.50–1.83)
Beach walking or jogging						
No	100 (22)	44 (19)	1.0	99 (22)	37 (24)	1.0
Yes	362 (78)	182 (81)	0.93 (0.61–1.42)	353 (78)	118 (76)	0.79 (0.50–1.25)
Wetlands walking or jogging						
No	388 (86)	190 (84)	1.0	379 (85)	126 (81)	1.0
Yes	67 (15)	36 (16)	1.13 (0.70–1.83)	66 (15)	29 (19)	1.20 (0.71–2.01)
Bushwalking						
No	339 (74)	172 (76)	1.0	332 (74)	120 (77)	1.0
Yes	119 (26)	54 (24)	0.73 (0.49–1.09)	116 (26)	35 (23)	0.70 (0.44–1.10)
Golf						
No	368 (81)	186 (82)	1.0	360 (81)	132 (85)	1.0
Yes	89 (19)	40 (18)	0.71 (0.46–1.11)	87 (19)	23 (15)	0.65 (0.38–1.10)
Sport on oval or field						
No	417 (91)	206 (91)	1.0	408 (91)	140 (90)	1.0
Yes	39 (9)	20 (8)	0.83 (0.46–1.51)	38 (9)	15 (10)	0.96 (0.49–1.85)
Swimming in lakes and rivers						
No	435 (95)	212 (94)	1.0	425 (95)	145 (94)	1.0
Yes	21 (5)	14 (6)	1.36 (0.65–2.84)	21 (5)	10 (6)	1.21 (0.54–2.74)
Sailing						
No	431 (95)	215 (95)	1.0	421 (94)	146 (94)	1.0
Yes	25 (5)	11 (5)	0.79 (0.38–1.67)	25 (6)	9 (6)	0.99 (0.44–2.20)
Outdoor barbecue						
No	215 (47)	91 (40)	1.0	211 (47)	68 (44)	1.0
Yes	242 (53)	135 (60)	1.19 (0.84–1.67)	236 (53)	87 (56)	1.08 (0.74–1.58)
Any reported outdoor activities‡						
No outdoor activities	18 (4)	18 (7)	1.0	16 (3)	16 (9)	1.0
Yes, any of the above or other outdoor activities reported	463 (96)	226 (93)	0.34 (0.17–0.68)	453 (97)	155 (91)	0.24 (0.11–0.52)
Outdoor activities in warmer months, d						
1, lowest tertile including none	138 (29)	108 (44)	1.0	131 (28)	72 (42)	1.0
2	149 (31)	87 (36)	0.64 (0.44–0.94)	146 (31)	52 (30)	0.57 (0.37–0.89)
3, highest tertile	194 (40)	49 (20)	0.30 (0.20–0.46)	192 (41)	47 (27)	0.43 (0.28–0.67)
Outdoor activities in cooler months, d						
1, lowest tertile including none	138 (29)	100 (41)	1.0	130 (28)	65 (38)	1.0
2	149 (31)	88 (36)	0.71 (0.49–1.05)	147 (31)	54 (32)	0.67 (0.43–1.05)
3, highest tertile	194 (40)	56 (23)	0.38 (0.25–0.58)	192 (41)	52 (30)	0.52 (0.34–0.81)

*Values are no. (%) except as indicated. aOR, adjusted odds ratio, NA, not applicable.
†Adjusted for age and sex.
‡Reported outdoor activities were beach walking or jogging, wetlands walking or jogging, bushwalking, golf, sport on oval or field, swimming in lakes and rivers, sailing, and outdoor barbecue, but not gardening.

Appendix Table 10. Potentially protective behaviors according to case and control population status and associations with Buruli ulcer*

	All participants			Residents only		
Behaviors	Controls	Cases	aOR† (95% CI)	Controls	Cases	aOR† (95% CI)
No. participants	481	245	NA	469	171	NA
Covers pre-existing wounds with dressing						
Never/Sometimes/Other	327 (67)	176 (72)	1.0	317 (68)	119 (70)	1.0
Usually/Always	154 (32)	69 (28)	0.99 (0.70–1.41)	152 (32)	52 (30)	1.06 (0.71–1.57)
Timeliness of tending to cuts and scratches from outdoors						
Leaves to heal naturally, or other response	185 (38)	126 (51)	1.0	178 (38)	82 (48)	1.0
Eventually (wash and dressing or antiseptic)	178 (37)	80 (33)	0.74 (0.51–1.05)	174 (37)	62 (36)	0.81 (0.55–1.21)
Immediately (wash and dressing or antiseptic)	118 (25)	39 (16)	0.56 (0.36–0.87)	117 (25)	27 (16)	0.54 (0.32–0.91)
Tending to cuts/scratches immediately (binary)						
No	363 (75)	206 (84)	1.0	352 (75)	144 (84)	1.0
Yes (usually/always)	118 (25)	39 (16)	0.65 (0.43–0.98)	117 (25)	27 (16)	0.60 (0.37–0.97)
Washes hands after outdoor activity						
Never/Sometimes	26 (6)	23 (10)	1.0	24 (5)	17 (10)	1.0
Usually/Always	435 (94)	213 (90)	0.62 (0.34–1.15)	426 (95)	148 (90)	0.53 (0.27–1.03)
Showers after outdoor activity						
Never/Sometimes	385 (80)	194 (79)	1.0	374 (80)	140 (82)	1.0
Usually/Always	96 (20)	51 (21)	0.94 (0.63–1.40)	95 (20)	31 (18)	0.80 (0.50–1.27)
Insect repellent use in warm months						
Never	99 (20.63)	78 (31.84)	1.0	97 (21)	57 (33)	1.0
Occasionally/Usually/Always	381 (79.38)	167 (68.16)	0.62 (0.43–0.89)	371 (79)	114 (67)	0.56 (0.38–0.84)
Clothing covering arms and legs						
Never/sometimes/seasonally	296 (63)	173 (72)	1.0	288 (63)	114 (68)	1.0
Usually/always, either arms or legs	76 (16)	38 (16)	0.92 (0.59–1.44)	74 (16)	33 (20)	1.12 (0.69–1.80)
Usually/always, both arms and legs	96 (21)	28 (12)	0.59 (0.36–0.95)	94 (21)	20 (12)	0.61 (0.35–1.05)
Gardening Gloves						
Never	79 (18.37)	59 (27.19)	1.0	79 (19)	39 (25)	1.0
Sometimes/usually/always	351 (81.63)	158 (72.81)	0.71 (0.47–1.07)	342 (81)	117 (75)	0.79 (0.50–1.24)
Closed shoes outside, warmer months						
Never/sometimes	280 (58)	147 (61)	1.0	273 (58)	96 (57)	1.0
Usually/always	199 (42)	95 (39)	0.99 (0.70–1.39)	194 (42)	73 (43)	1.14 (0.77–1.67)
No. multiple examined behaviors						
0–1	21 (4)	30 (12)	1.0	20 (4)	21 (12)	1.0
2–3	195 (41)	106 (43)	0.41 (0.22–0.76)	191 (41)	67 (39)	0.34 (0.17–0.67)
4–5	193 (40)	92 (38)	0.39 (0.21–0.75)	186 (40)	70 (41)	0.39 (0.19–0.77)
>6	72 (15)	17 (7)	0.22 (0.10–0.48)	72 (15)	13 (8)	0.20 (0.08–0.48)
No. multiple behaviors identified as protective						
None	61 (13)	50 (20)	1.0	60 (13)	38 (22)	1.0
1	224 (47)	132 (54)	0.75 (0.48–1.16)	217 (46)	83 (49)	0.62 (0.38–1.003)
2	141 (29)	49 (20)	0.50 (0.30–0.83)	137 (29)	39 (23)	0.49 (0.28–0.85)
3	55 (11)	14 (6)	0.38 (0.19–0.78)	55 (12)	11 (6)	0.36 (0.16–0.78)

*Values are no. (%) except as indicated. aOR, adjusted odds ratio, NA, not applicable.
†Adjusted for age and sex.

Appendix Table 11. Sensitivity analysis of BCG vaccination and Buruli ulcer case status restricted to participants who were 47–70 years of age and potentially eligible for routine BCG vaccination in Australia*

	All participants			Residents only		
Status	Controls	Cases	aOR† (95% CI)	Controls	Cases	aOR† (95% CI)
No. participants	260	115	NA	260	115	NA
BCG vaccination						
No	50 (19.23)	30 (26.09)	1.0	50 (19.23)	24 (30.38)	1.0
Yes	139 (53.46)	45 (39.13)	0.57 (0.32–1.03)	136 (53.33)	29 (36.71)	0.44 (0.23–0.85)
Unsure	71 (27.31)	40 (34.78)	0.97 (0.52–1.82)	69 (27.06)	26 (32.91)	
BCG vaccination, unsure assumed yes						
No	50 (19.23)	30 (26.09)	1.0	50 (19.61)	24 (30.38)	1.0
Yes/unsure	210 (80.77)	85 (73.91)	0.71 (0.41–1.22)	205 (80.39)	55 (69.62)	0.56 (0.31–1.02)

*Values are no. (%) except as indicated. Participants in this age range would be 12 years old from ≈1960–1983; therefore, they were covered during the years when routine vaccination was reported in many states in Australia. The assumption that participants who were unsure of their status would likely have had the vaccination as part of routine vaccination might involve some misclassification. Participants might have resided in other countries or states in Australia without routine BCG vaccination. aOR, adjusted odds ratio, NA, not applicable.
†Adjusted for age and sex.

Appendix Table 12. Representativeness of case-control participant samples*

Characteristics	Controls, no. (%)	Weighted population,† %	Ratio‡	Cases, no. (%)	Notified cases, no. (%)	% Total notified cases§	Ratio¶
No. participants	481	NA	NA	245	NA	NA	NA
Age group, y							
18–39	38 (7.9)	29.5	0.27	35 (14.3)	134 (24.4)	26%	0.59
40–59	125 (26.0)	32.7	0.80	68 (27.8)	154 (28.0)	44%	0.99
60–79	278 (57.8)	29.8	1.94	123 (50.2)	204 (37.1)	60%	1.85
≥80	40 (8.3)	8.1	1.03	19 (7.8)	58 (10.6)	33%	0.74
Sex							
F	266 (55.3)	51.3	1.08	104 (42.5)	228 (41.5)	46%	1.02
M	215 (44.7)	48.7	0.92	141 (57.6)	321 (58.4)	44%	0.99
Not stated	NA	NA	NA	NA	1 (0.2)	NA	NA

*NA, not applicable.
†Local government area–weighted population estimates according to age group and sex were derived by using Australian Bureau of Statistics Estimated Resident Population, which, for each of the relevant areas, were weighted to the percentages in our control sample. Control percentages: Mornington Peninsula (64.86%), Frankston (9.36%), Bayside (8.32%), Kingston (4.57%), Greater Geelong (7.90%), Queenscliffe (3.53%), and Surf Coast (1.46%).
‡Ratio of percentages by age group (controls to weighted population estimate).
§Notified case-patients participating in included sample, group participation rate.
¶Ratio of percentages by age group (included cases to notified cases).

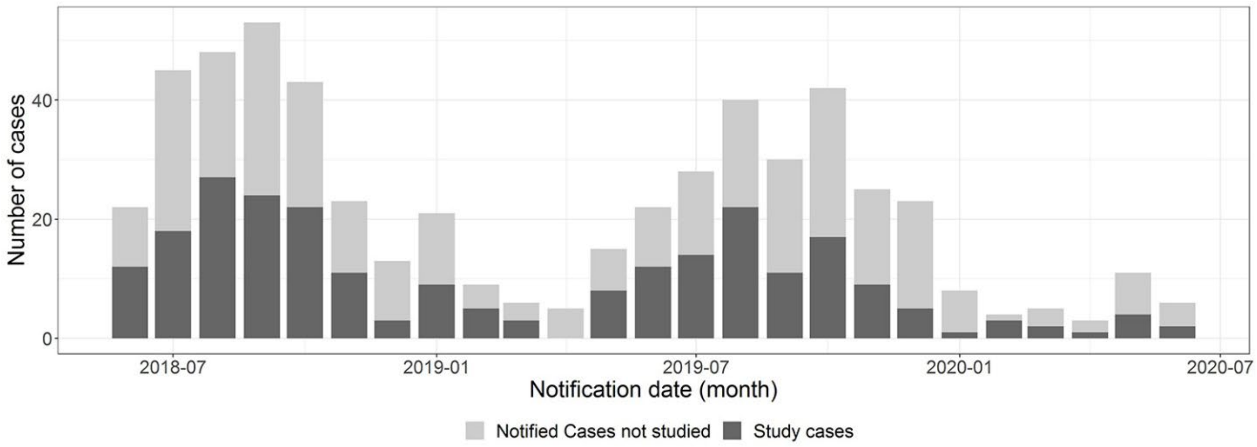

Appendix Figure 1. Number of patients with Buruli ulcer reported to the Department of Health in Victoria, Australia, during June 2018–June 2020. Month and year of case notification for Buruli ulcer patients (dark gray) participating in this study and all other notified Buruli ulcer cases (light gray). Cases were reported from Buruli ulcer–endemic locations in Victoria.

	Tends to cuts and scratches immediately (binary)	Covers pre-existing wounds	Washes hands after outdoor activity	Showers after outdoor activity	Insect repellent (warmer months)	Gardening gloves	Clothing covers arms and/or legs	Closed Shoes outside (warmer months)	*Drinking filtered town water*
Tends to cuts and scratches immediately									
Covers pre-existing wounds	0.56								
Washes hands after outdoor activity	0.48	0.42							
Showers after outdoor activity	0.21	0.26	0.37						
Insect repellent (warmer months)	0.20	0.25	0.31	0.19					
Gardening gloves	0.40	0.46	0.44	0.06	0.22				
Clothing covers arms and/or legs	0.34	0.40	0.33	0.11	0.14	0.32			
Closed shoes outside (warmer months)	0.09	0.08	0.08	-0.02	0.05	0.08	0.30		
*Drinking filtered town water**	0.15	0.12	0.14	0.06	0.12	0.09	-0.01	0.07	

Appendix Figure 2. Polychoric correlations between potential protective behaviors against Buruli ulcer in all case-control study participants from the study areas in Victoria, Australia. Drinking filtered town water (asterisk) was examined as a potential protective factor; because of lack of correlations with other behaviors and low factor loadings in a preliminary exploratory 2-factor model, this factor was not included in the final analysis for assessing the factor structure. Numbers are correlation coefficients between factors.

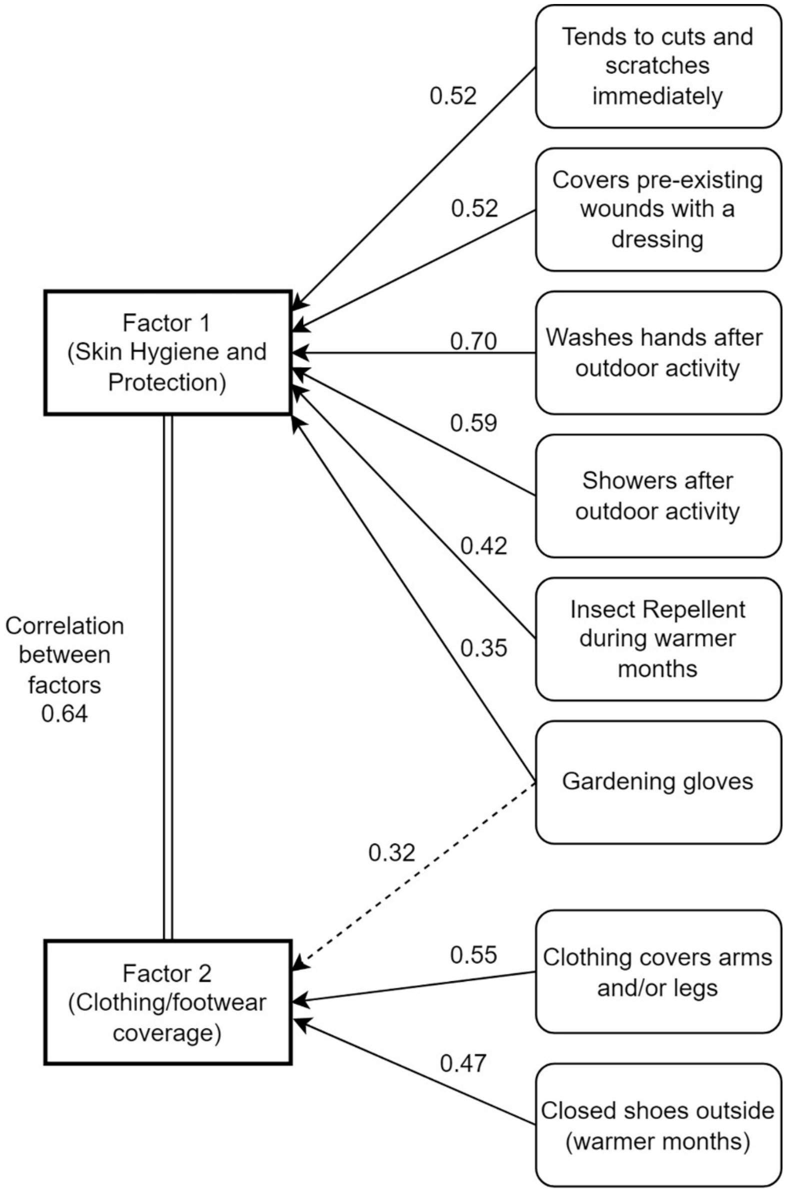

Appendix Figure 3. Factor structure and rotated factor loadings from exploratory factor analysis of the clustering of potentially protective behaviors, examined in relation to Buruli ulcer prevention in Victoria, Australia. Numbers are correlation coefficients between factors.
